# Creativity and the default network: A functional connectivity analysis of the creative brain at rest^☆^

**DOI:** 10.1016/j.neuropsychologia.2014.09.019

**Published:** 2014-11

**Authors:** Roger E. Beaty, Mathias Benedek, Robin W. Wilkins, Emanuel Jauk, Andreas Fink, Paul J. Silvia, Donald A. Hodges, Karl Koschutnig, Aljoscha C. Neubauer

**Affiliations:** a Department of Psychology, University of North Carolina at Greensboro, USA; b Department of Psychology, University of Graz, Austria; c Music Research Institute, University of North Carolina at Greensboro, USA; d Joint School of Nanoscience and Nanoengineering, Greensboro, USA

**Keywords:** Creative cognition, Default mode network, Divergent thinking, Resting-state functional connectivity, Inferior frontal gyrus

## Abstract

The present research used resting-state functional magnetic resonance imaging (fMRI) to examine whether the ability to generate creative ideas corresponds to differences in the intrinsic organization of functional networks in the brain. We examined the functional connectivity between regions commonly implicated in neuroimaging studies of divergent thinking, including the inferior prefrontal cortex and the core hubs of the default network. Participants were prescreened on a battery of divergent thinking tests and assigned to high- and low-creative groups based on task performance. Seed-based functional connectivity analysis revealed greater connectivity between the left inferior frontal gyrus (IFG) and the entire default mode network in the high-creative group. The right IFG also showed greater functional connectivity with bilateral inferior parietal cortex and the left dorsolateral prefrontal cortex in the high-creative group. The results suggest that the ability to generate creative ideas is characterized by increased functional connectivity between the inferior prefrontal cortex and the default network, pointing to a greater cooperation between brain regions associated with cognitive control and low-level imaginative processes.

## Highlights

• Participants completed divergent thinking tasks and resting-state fMRI
• Higher creative ability was related to greater rs-fc in the IFG and default network
• Seed-to-voxel analysis further revealed greater rs-fc in the IFG and DLPFC
• Controlled and spontaneous processes may cooperate more in the creative brain

## 1 Introduction

The neuroscience of creativity is a topic of increasing interest but little empirical consensus. Recent literature reviews have been largely inconclusive on the brain basis of creative thought (Arden et al., 2010, Dietrich and Kanso, 2010, Sawyer, 2011), raising questions about whether creativity can be isolated to discrete regions in the brain (Abraham, 2013, Jung et al., 2013). Despite the lack of consensus in the literature at large, an emerging literature on divergent thinking, a central component of general creative ability, has yielded a relatively consistent pattern of results (Abraham et al., 2012, Benedek et al., 2014, Fink et al., 2009, Fink and Benedek, 2014, Gonen-Yaacovi et al., 2013). Such work points to an important role of the inferior prefrontal cortex—regions associated with controlled memory retrieval (Badre & Wagner, 2007) and central executive processes (Aron, 2007)—and the default mode network—regions associated with internally-directed attention and spontaneous cognition (Andrews-Hanna, 2012).

But because these regions also correspond to large-scale networks with seemingly opposing functional roles (Fox et al., 2005), the extent to which they cooperate to support creative thought remains unclear. Does activation of disparate regions during divergent thinking reflect isolated contributions to the same process, or does such activity reflect the underlying presence of a functionally interconnected network? The present research sought to address the relationship between divergent thinking ability and resting-state functional connectivity. To this end, we conducted a series of functional connectivity analyses in individuals of high- and low-creative ability, and examined the extent to which brain regions associated with different modes of attention and cognition exhibit greater functional connectivity in the highly creative brain.

### 1.1 The cognitive and neural basis of divergent thinking

Divergent thinking has a long tradition in the creativity literature. Guilford (1950) introduced the construct and advanced a mode of assessment that remains widely used in modern research (Kaufman, Plucker, & Baer, 2008). The most common divergent thinking assessment is the alternate uses task, which involves producing novel uses for common objects (e.g., a brick). A central virtue of such tasks is their predictive power: a longitudinal study of divergent thinking ability in school-aged children, for example, found that the top performers eventually lead highly successful careers in the arts and sciences (Plucker, 1999, Torrance, 1988). Other work has since shown that divergent thinking predicts both the quantity of self-reported creative achievements (Jauk, Benedek, & Neubauer, 2014) and the quality of expert-rated creative performances (Beaty, Smeekens, Silvia, Hodges, & Kane, 2013).

The predictive power of divergent thinking tasks has fueled empirical interest in the cognitive basis of creative thought. An increasing body of research suggests that divergent thinking involves the top-down control of attention and cognition. Much of this evidence comes from latent variable studies showing effects of higher-order cognitive abilities, such as fluid intelligence (Beaty et al., 2014, Nusbaum and Silvia, 2011), working memory capacity (Lee and Therriault, 2013, Süß et al., 2002), and verbal fluency (Benedek et al., 2012b, Silvia et al., 2013). Such abilities are hypothesized to support the creative process by providing the executive control needed to guide memory retrieval and inhibit salient but unoriginal ideas (Beaty and Silvia, 2012, Beaty and Silvia., 2013, Benedek et al., 2014, Gilhooly et al., 2007, Silvia et al., 2014). Nevertheless, the role of cognitive control in creative thought remains controversial, as other work supports a defocused attention account of creativity (Takeuchi et al., 2011) and a limit to the correlation between creativity and intelligence (Jauk et al., 2013, Jung et al., 2009).

Behavioral evidence for the role of executive processes in divergent thinking has received support from electroencephalogram (EEG) and functional magnetic resonance imaging (fMRI) research. Several studies report task-related activation in brain regions associated with cognitive control, such as the inferior frontal gyrus (IFG) and inferior parietal cortex (Abraham et al., 2012, Benedek et al., 2014, Chrysikou and Thompson- Schill, 2011, Fink et al., 2009, Fink and Benedek, 2014). Fink et al. (2009), for example, conducted an fMRI study with a battery of divergent thinking tasks that varied in terms of the creativity-related demands required. Tasks with a high-creativity demand required the generation of novel uses for common objects—the classic divergent thinking task—and tasks with a low-creativity demand simply required the generation of typical object characteristics. Compared to tasks with low-creativity demands, performance on tasks with high-creativity demands was associated with increased activation of the left angular gyrus and decreased activation in right inferior parietal cortex. Moreover, regardless of the task demands, idea generation was related to increased activation of the left IFG, anterior cingulate cortex, and the precentral gyrus. Fink and colleagues interpreted their results as evidence for a role of controlled memory retrieval and internal attention in divergent thinking.

The inferior prefrontal cortex is commonly implicated in neuroimaging studies of divergent thinking (Abraham et al., 2012, Benedek et al., 2014, Chrysikou and Thompson- Schill, 2011, Kleibeuker et al., 2013, Vartanian et al., 2013). A recent meta-analysis, which included 34 fMRI studies and a variety of creativity tasks, found that the left IFG was among the most strongly activated regions during tasks involving idea generation (Gonen-Yaacovi et al., 2013). Benedek et al. (2014) further highlighted the role of the inferior prefrontal cortex by showing that activation of the left IFG increased with the creative quality of divergent thinking responses produced during functional imaging. In a related line of work, an fMRI study of figurative language production, employing divergent thinking tasks that required producing metaphors and synonyms, found that the left IFG was associated with the process of idea generation (Benedek et al., 2014). Taken together, an emerging literature provides support for the role of the inferior prefrontal cortex in creative thought, a region associated with controlled semantic retrieval (Badre & Wagner, 2007) and pre-potent response inhibition (Aron, 2007, Dodds et al., 2011, Rae et al., 2014).

But the notion that divergent thinking is solely a controlled cognitive process is not fully consistent with the literature, as many of the same studies that report activation in brain regions associated with controlled cognitive processes also report activation in regions associated with spontaneous processes. Specifically, regions within the default mode network (DMN) have been reported in several recent studies of divergent thinking (Benedek et al., 2014, Ellamil et al., 2012, Fink et al., 2009, Fink et al., 2012; Gonen-Yaacovi et al., 2013, Jung et al., 2010, Takeuchi et al., 2012, Wei et al., 2014). The DMN includes the medial prefrontal cortex (mPFC), the posterior cingulate cortex (PCC) and the adjacent the precuneus, and bilateral inferior parietal lobes (IPL; Fox et al., 2005, Gusnard and Raichle, 2001). This network has been shown to consistently decrease in activation when an external task is presented and increase in the absence of external task demands (Buckner et al., 2008, Raichle et al., 2001). Since the initial discovery of the DMN, a large body of research has sought to elucidate its underlying function. Such work implicates a wide range of mental phenomena, including episodic future thinking (Schacter et al., 2012), mental simulation (Hassabis & Maguire, 2007), perspective-taking (Buckner & Carroll, 2007), and mind wandering (Andrews-Hanna, 2012, Christoff et al., 2009). Creativity researchers have recently begun to speculate about the potential role of the DMN in creative thought. For example, default mode activity has been hypothesized to underlie blind-variation and selective-retention processes (Jung et al., 2013) and internally-directed attention during divergent thinking (Benedek et al., 2011, Benedek et al., 2014, Fink and Benedek, 2014).

Further evidence for the role of the DMN in divergent thinking comes from two recent studies reporting associations between divergent thinking and functional connectivity in default mode regions (Takeuchi et al., 2012, Wei et al., 2014). Both studies employed resting-state functional magnetic resonance imaging (rs-fMRI), a technique that measures spontaneous temporal correlations between blood oxygen level-dependent (BfMOLD) signals in the brain. Takeuchi et al. (2012) assessed divergent thinking ability outside of the scanner and then measured rs-fMRI. A functional connectivity analysis, with the mPFC specified as a seed region of interest, found that the strength of connectivity between the mPFC and PCC increased with divergent thinking scores. Wei et al. (2014) further demonstrated an association between divergent thinking and default mode regions, reporting increased functional connectivity between the mPFC and the middle temporal gyrus. Moreover, a recent meta-analysis found divergent thinking-related activation in regions of the DMN, including the PCC and bilateral inferior parietal cortex (Gonen-Yaacovi et al., 2013). Taken together, a growing body of evidence suggests that divergent thinking is related to functional activation of the DMN—regions associated with spontaneous cognition—and the inferior prefrontal cortex—regions associated with cognitive control.

### 1.2 The present research

Although recent research supports the notion that divergent thinking recruits controlled cognitive processes in the inferior prefrontal cortex, the literature also implicates regions of the DMN, indicating that creative thought may also involve uncontrolled processes. The extent to which these seemingly disparate brain regions cooperate to support creative thought thus remains unclear. Because the brain is a highly complex system composed of functionally interconnected neural networks (Bullmore and Sporns, 2009, Fox et al., 2005, van den Heuvel and Hulshoff Pol, 2010), the interaction between individual regions is critical to understanding how cognitive processes like divergent thinking unfold.

The present research sought to address the question of whether discrete brain regions linked with divergent thinking show increased functional connectivity in people of higher creative ability. To this end, we analyzed resting-state functional connectivity in participants of both high- and low-divergent thinking ability. Participants completed a battery of divergent thinking tasks in the lab and subsequently underwent resting-state functional imaging. In light of past research, we were particularly interested in exploring functional connectivity between the inferior prefrontal cortex and the core hubs of the DMN. This approach allowed us to determine whether regions within default mode and cognitive control networks exhibit stronger functional connectivity in highly creative individuals.

## 2 Method

### 2.1 Participants

All participants were right-handed, native-German speakers with corrected-to-normal vision, and no history of CNS-affecting drugs or neurological disease. Participants provided written consent and were paid for their time. The study was approved by the local ethics committee at the University of Graz.

### 2.2 Behavioral assessment and pre-screening procedure

The sample described here was taken from a larger pool of subjects that participated in previous MRI research at the University of Graz (*n*=91). Participants were prescreened for this study based on their performance on a battery of six computerized divergent thinking tasks. We used a between-groups approach to characterize the intrinsic functional architecture of individuals of high-creative ability. Participants at the top 33% of the sample were assigned to the *high-creative* group, and participants at the bottom 33% of the sample were assigned to the *low-creative* group; the middle 33% was not included in the analysis. As described below, the two groups were carefully selected and matched to control for several variables associated with creative ability.

The divergent thinking test battery consisted of three alternate uses tasks and three instances tasks (Kaufman et al., 2008). The alternate uses tasks required participants to generate creative uses for three common objects: a *can*, a *knife*, and a *hairdryer*. The instances tasks required participants to generate creative solutions to the three problems: *What can make noise*?, *What can be elastic*?, and *What can be used for speedy travel*?

After each task, participants were presented with their list of responses and asked to rank them for creative quality. Responses to all six tasks were later scored by three trained raters using the subjective scoring method (Benedek et al., 2013, Christensen et al., 1957, Silvia et al., 2008), an approach grounded in the consensual assessment technique of creativity assessment (Amabile, 1982). The three raters were trained to score responses for creative quality, using a 1 (*not at all creative*) to 4 (*very creative*) scale. We applied the Top 2 scoring procedure (Silvia et al., 2008) by selecting the two most creative responses indicated by participant rankings and averaged the three raters׳ scores. The creativity scores of the entire sample of participants were then rank-ordered.

We also administered a series of personality questionnaires and cognitive tasks. Because divergent thinking is associated with intelligence (Beaty and Silvia, 2012, Benedek et al., 2012, Jauk et al., 2014, Nusbaum and Silvia, 2011) and the personality trait openness to experience (Feist, 1998, McCrae, 1987, Silvia et al., 2009), participants completed a battery of intelligence tests from the Intelligence Structure Battery (Arendasy et al., 2004) and the Big-Five Structure Inventory (Arendasy, Sommer, & Feldhammer, 2011; see Jauk et al., 2014 for more information on the tasks and questionnaires). Participants also completed demographic questionnaires.

We carefully matched the groups by iteratively removing participants until they were matched on intelligence, personality, age, and gender (see Table 1). This procedure results in two well-matched groups—*high-creative* (*n*=12; mean age=27.33, *SD*=9.26; 7 women) and *low-creative* (*n*=12; mean age=31.40, *SD*=9.05; 7 women). A series of between-groups *t*-tests revealed that these groups did not differ significantly in terms of intelligence (*p*=.203), openness to experience (*p*=.101), age (*p*=.287), or gender (*p*=1.00). The high- and low-creative groups were, however, substantially different in terms of divergent thinking ability (*p*<.001). The two groups were thus equated on several variables associated with creativity, permitting an analysis of group differences in functional connectivity related to divergent thinking ability.

**Table 1:** Demographic and behavioral data for the high- and low-creative groups.

	**High-creative**	**Low-creative**	***p***
DT-AU: Task 1	2.32 (.27)	1.98 (.30)	.010
DT-AU: Task 2	2.30 (.45)	1.85 (.31)	.010
DT-AU: Task 3	2.28 (.15)	1.90 (.30)	.001
DT-IN: Task 1	2.10 (.33)	1.86 (.24)	.055
DT-IN: Task 2	2.37 (.12)	1.81(.81)	.003
DT-IN: Task 3	2.38 (.23)	1.89 (.28)	.002
DT: Composite Avg.	2.27 (.12)	1.88 (.10)	<.001
IQ	115.81 (16.34)	106.76 (17.40)	.203
FFI: Neuroticism	.14 (.61)	.15 (.82)	.958
FFI: Extraversion	−.13 (.94)	.33 (.90)	.227
FFI: Openness to experience	−.33 (.67)	.13 (.67)	.101
FFI: Agreeableness	.17 (1.02)	.01 (.76)	.667
FFI: Conscientiousness	.01 (.82)	−.23 (.87)	.466
Age	27.33 (9.26)	31.40 (9.05)	.287
Gender	7 women; 5 men	7 women; 5 men	1.00

*Note.* The table displays group means and standard deviations (in parentheses). Independent sample *t*-tests were computed and corresponding p-values are listed in the far right column. Personality variables are Item Response Theory (IRT) scores. DT-AU=Divergent Thinking-Alternate Uses; DT-IN=Divergent Thinking-Instances; FFI=Five Factor Inventory; IQ=Intelligence Quotient.

### 2.3 Functional MRI data acquisition

Participants were scanned using a 3T Siemens Skyra system (Siemens Medical Systems, Erlangen, Germany) with a 32-channel head coil. BOLD-sensitive T2^⁎^-weighted functional images were acquired using a single shot gradient-echo EPI pulse sequence (TR=2500 ms, TE=27 ms, flip angle=90°, 32 axial slices, 4.0×4.0×4.0 mm^3^, distance factor 25%, FoV=256×256 mm^2^, interleaved slice ordering) and corrected online for head motion. The first two volumes were discarded to allow for T1 equilibration effects. Head motion was restricted using firm padding that surrounded the head. Data were acquired for five minutes while participants rested with their eyes closed. Following functional imaging, a high resolution T1 scan was acquired for anatomic normalization. Imaging data were then slice-time corrected and realigned using the Statistical Parametric Mapping (SPM) 8 package (Wellcome Institute of Cognitive Neurology, London). Functional volumes were coregistered and resliced to a voxel size of 2 mm³, normalized to the MNI template brain (Montreal Neurological Institute), and smoothed with an 8 mm^3^ isotropic Gaussian kernel.

### 2.4 Functional connectivity analysis

Functional connectivity analysis was implemented in MATLAB using the CONN toolbox (http://www.nitrc.org/projects/conn; Whitfield-Gabrieli & Nieto-Castanon, 2012). For each participant, CONN implemented the CompCor method (Behzadi, Restom, Liau, & Liu, 2007) to identify principal components associated with segmented white matter (WM) and cerebrospinal fluid (CSF). WM, CSF, and realignment parameters were entered as confounds in a first-level analysis (Behzadi et al., 2007), and the data were band-pass filtered to .008 Hz–.09 Hz. CompCor addresses the confounding effects of subject movement without affecting intrinsic functional connectivity (Chai Nieto-Castanon, Ongur, & Whitfield-Gabrieli, 2012), thus global signal was not regressed.

We then conducted a region of interest (ROI) analysis and a seed-to-voxel analysis. The ROI-to-ROI analysis allowed us to test hypotheses regarding the functional connectivity between the inferior prefrontal cortex and the DMN. We specified six 10 mm spherical clusters with peak- coordinates based on a reliability analysis of resting-state data (cf. Whitfield-Gabrieli & Nieto-Castanon, 2012). Two ROIs were located in bilateral inferior prefrontal cortex—left IFG (−34, 24, −11) and right IFG (36, 24 −11)—corresponding to Brodmann area 47. The other four ROIs were located in the DMN—mPFC (0, 54, −8), PCC (0,–56, 28), left IPL (−42,–68, 38), and right IPL (48,–60, 38). CONN computed temporal correlations between the BOLD signals in the two seed ROIs—bilateral IFG—and four target ROIs in the DMN. This procedure was applied to both the high- and low-creative groups. *t*-tests and Fisher׳s Z-transformed correlations were computed to analyze differences in functional connectivity between the seed and target ROIs across groups. ROI-to-ROI results are reported when significant at a level of *p* <.05 false discovery rate (FDR) corrected (Chumbley, Worsley, Flandin, & Friston, 2010).

Next, we conducted a seed-to-voxel analysis. This allowed us to explore whether bilateral IFG was differentially connected to other brain regions outside of the DMN in the highly-creative group. Using the same seed ROIs in bilateral IFG defined above, temporal correlations were computed between these seeds and all other voxels in the brain. *t*-tests and Fisher׳s Z-transformed correlations were used to compute differences in functional connectivity between the high- and low-creative groups. Seed-to-voxel results are reported when significant at a voxelwise threshold of level of *p*<.001 uncorrected and a cluster-level threshold of *p*<.05 FDR corrected. All coordinates reported below refer to peak activations in anatomical MNI space.

## 3 Results

### 3.1 ROI-to-ROI analysis

We began with the ROI-to-ROI analysis to examine the functional connectivity between bilateral IFG and default mode regions. This analysis revealed significantly stronger connectivity in the high-creative group in the left IFG and the entire default mode network: left IFG-left IPL (*t*=3.00, *p*=.008), left IFG-PCC (*t*=3.34, *p*=.005), left IFG-right IPL (*t*=3.75, *p* =.004), and left IFG-mPFC (*t*=2.38, *p*=.026). This suggests that the left IFG is more strongly connected to the DMN in participants with high divergent thinking ability.

The high-creative group also showed significantly stronger connectivity between right IFG and bilateral IPL: right IFG-left IPL (*t*=2.76, *p*=.022) and right IFG-right IPL (*t*=3.36, *p*=.011). The highly-creative group did not, however, show greater connectivity between the right

IFG and the mPFC or the PCC at a conservative level of significance (i.e., *p*<.05 FDR corrected), nor did this pattern emerge at a less conservative significance level (*p*<.05 uncorrected). Thus, compared to the low-creative group, the high-creative group showed different patterns of connectivity between the right IFG and regions of the DMN.

### 3.2 Seed-to-voxel analysis

Our ROI-to-ROI analysis found increased functional connectivity between bilateral IFG and default mode regions in highly-creative participants. We then proceeded to a seed-to-voxel analysis. This allowed us to extend the ROI-to-ROI analysis by determining whether bilateral IFG showed greater connectivity with regions outside of the DMN.

**Fig. 1:**
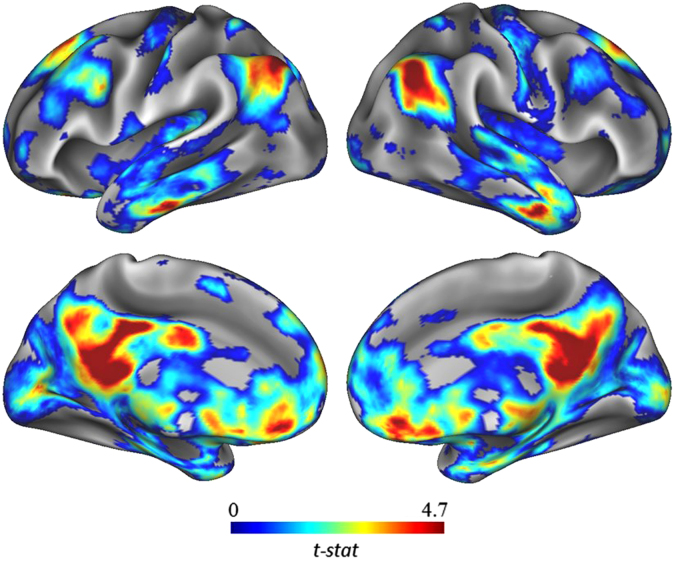
Group contrasts of seed-to-voxel connectivity maps with left IFG seed showing increased functional connectivity associated with greater divergent thinking ability.

We thus performed a whole-brain, between-group seed-to-voxel analysis to examine potential group differences in functional connectivity between the left IFG and all other voxels in the brain (see Fig. 1). In line with previous analysis, the high-creative group showed significantly stronger connectivity between the left IFG and a large cluster of voxels in posterior cingulate cortex (*k*=791; −6, −34, 34). The high-creative group also showed stronger connectivity between the left IFG and a cluster of voxels in right inferior parietal cortex (*k*=257; 48, −60, 38). The highly-creative group also showed stronger connectivity between the left IFG and a cluster of voxels in left inferior parietal cortex (*k*=80; −38, −76, 48). Taken together, the results of the seed-to-voxel largely confirm the results of the ROI-to-ROI results reported above.

**Fig. 2:**
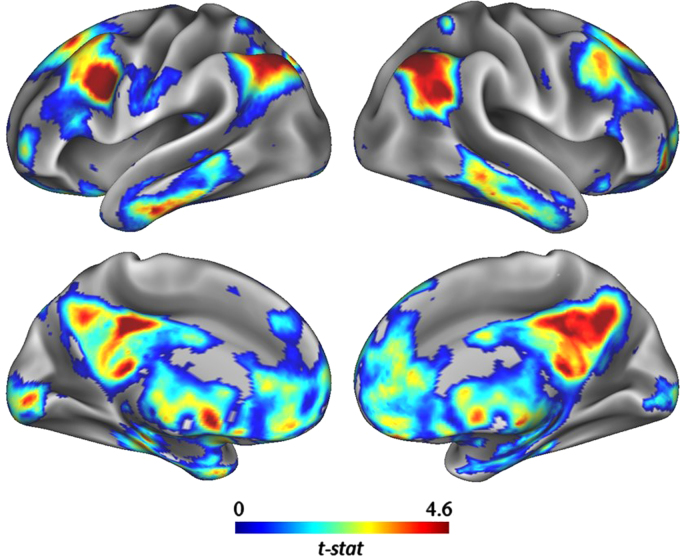
Group contrasts of seed-to-voxel connectivity maps with right IFG seed showing increased functional connectivity associated with greater divergent thinking ability.

Our final analysis contrasted the functional connectivity between the high- and low-creative groups in the right IFG and the rest of the brain (see Fig. 2). In line with the ROI-to ROI analysis, the high-creative group showed significantly stronger connectivity between the right IFG and a cluster of voxels in the right inferior parietal lobe (*k*=154; 46, −68, 50). Novel to the current analysis, the high-creative group showed stronger connectivity between the right IFG and a cluster of voxels in left dorsolateral prefrontal cortex (*k*=86; −36, 4 and 30).

## 4 Discussion

The present study explored divergent thinking-related functional connectivity between inferior prefrontal cortex and the DMN. We used resting-state functional imaging to analyze intrinsic connectivity differences in groups of high- and low-divergent thinking ability. Resultsrevealed increased functional connectivity between seed regions in inferior prefrontal cortex (bilateral IFG) and the DMN (mPFC, PCC, and bilateral IPL) associated with greater divergentthinking ability. This research extends past work by demonstrating that discrete brain regions commonly linked with divergent thinking in the creativity literature are actually more strongly functionally connected in highly creative individuals. The results further suggest that divergent thinking ability involves a greater cooperation between brain regions associated with controlled and spontaneous cognitive processes.

A ROI-to-ROI analysis, contrasting groups of low- and high-divergent thinking ability, found greater functional connectivity between bilateral IFG and the DMN. This analysis revealed greater functional connectivity between the left IFG and all four default mode regions (i.e., mPFC, PCC, and bilateral IPL). Furthermore, the right IFG showed greater connectivity to bilateral IPL in the highly creative group, but not the mPFC and PCC. Taken together, these findings suggest that the ability to come up with creative ideas is associated with functional coupling between bilateral IFG and regions of the DMN.

A seed-to-voxel analysis extended the ROI-to-ROI analysis by exploring the extent to which bilateral IFG showed greater functional connectivity with other brain regions in the highly creative group. These results were largely in line with the prior analysis: the left and right IFG showed greater functional connectivity with clusters of voxels in several regions of the DMN. The left IFG seed was significantly more connected to a large cluster of voxels in cingulate cortex; a second cluster was found in the right inferior parietal cortex, which overlapped with the target ROI in inferior parietal cortex. This analysis further revealed greater connectivity between the left IFG seed and a cluster of voxels in left inferior parietal cortex, overlapping with the left angular gyrus.

The seed-to-voxel analysis also found that the right IFG seed was more strongly connected to right inferior parietal cortex. The right IFG seed was also more strongly connected to a cluster of voxels in left DLPFC—a region associated with controlled attention and working memory capacity (Curtis and D’Esposito, 2003, MacDonald et al., 2000)—suggesting enhanced cooperation between two brain areas linked with cognitive control in highly creative individuals. Recent research has reported that divergent thinking ability is related to increased functional connectivity among default mode regions (Takeuchi et al., 2012, Wei et al., 2014). The present findings extend this work by demonstrating that divergent thinking ability is also related to an increased functional coupling of executive control and default mode networks.

### 4.1 Creativity and cognitive control

The present study raises the intriguing possibility that creative thinking involves both controlled and spontaneous cognitive processes. But how might such seemingly opposing processes cooperate? Jung et al. (2013) proposed that divergent thinking-related activation of default mode and executive control networks correspond to blind variation and selective retention processes, respectively. The blind variation and selective retention (BVSR) theory is an evolutionary model of the creative process proposed by Campbell (1960) and extended by Simonton (1999). Jung and colleagues suggest that blind variation—an uncontrolled process that involves random conceptual combination—may occur in the DMN. Selective retention—a controlled process that involves evaluating blind variation activity—may occur in executive control regions of the brain. The BVSR model thus offers one possible explanation for the present results: increased functional connectivity between the DMN and bilateral IFG may reflect blind variation and selective retention processes working more closely together in the highly creative brain.

Other theories have sought to explain the role of attention in creativity. One compelling theory suggests that a “failure to deactivate” regions of the default network during tasks requiring focused external attention may characterize high creative ability (Takeuchi et al., 2011). Takeuchi and colleagues found that highly creative participants failed to suppress activity in the precuneus while engaging in a working memory task, suggesting that creativity may benefit from the coactivation of executive control and default mode networks. Moreover, a recent study on creative drawing found differential contributions of executive control and default networks during different stages of the drawing process (Ellamil et al., 2012). For example, regions of the default network were more strongly activated during idea generation, and regions of the executive control network were more strongly activated during idea evaluation. In addition, a functional connectivity analysis found increased coupling of executive control and default networks throughout the creative process, consistent with the notion that creativity requires flexible cognitive control (Zabelina & Robinson, 2010).

We propose a similar, but more general account of the present findings based on a controlled attention view of creative thought (Beaty and Silvia, 2012, Nusbaum and Silvia, 2011). Default network activity is associated with a wide range of imaginative processes, such as mind wandering (Andrews-Hanna, 2012, Christoff et al., 2009), mental simulation (Hassabis & Maguire, 2007), and episodic future thinking (Schacter et al., 2012). Activation of the DMN can hence be viewed as corresponding to a series of low-level, spontaneous processes with potential relevance to creative thought. Considering the present findings, then, divergent thinking-related functional connectivity between the inferior prefrontal cortex and the DMN may reflect the top-down control of bottom-up processes. In other words, cognitive control mechanisms in the inferior prefrontal cortex may be responsible for directing and monitoring spontaneous activity stemming from default mode activity.

Controlled attention appears to be particularly relevant to divergent thinking because salient, unoriginal ideas can impede the creative thought process. During an alternate uses task, for example, concepts that are strongly semantically associated with the prompt cue (e.g., brick) are often the first responses produced (e.g., “build a brick house”; Beaty and Silvia, 2012, Christensen et al., 1957). Cognitive control may support divergent thinking by inhibiting unoriginal ideas and shifting attention to different semantic categories (Nusbaum & Silvia, 2011). In the absence of sufficient cognitive control, divergent thinking can be compromised by an inability to exert control over the creative thought process and effectively move beyond pre-potent response tendencies (Benedek and Neubauer, 2013, Benedek et al., 2012, Gilhooly et al., 2007). The inferior prefrontal cortex may thus serve a range of supervisory, executive functions.

Recent research suggests that the frontoparietal control network, a large-scale network associated with cognitive control and decision-making processes (Vincent, Kahn, Snyder, Raichle, & Buckner, 2008), interacts with the DMN during mental simulation (e.g., autobiographical planning; Spreng & Schacter, 2012). Within the context of the present study, increased connectivity between the right IFG and inferior parietal cortex could reflect an underlying ability of highly creative people to exert top-down control over imaginative process arising from the DMN. The left IFG may provide further oversight by guiding search processes and evaluating candidate responses. The left IFG has been implicated in fMRI studies of divergent thinking (Benedek et al., 2014, Benedek et al., 2014, Fink et al., 2009) and controlled memory retrieval (Costafreda et al., 2006, Hirshorn and Thompson-Schill, 2006). Taken together, increased functional connectivity between the inferior prefrontal cortex and default mode regions may correspond to a greater ability of creative individuals to govern their imaginations, by executing complex search processes, inhibiting task-irrelevant information, and selecting ideas among a large set of competing alternatives.

### 4.2 Limitations and future directions

The present research found that divergent thinking ability was associated with increased resting-state functional connectivity between the inferior prefrontal cortex and the DMN. Our approach extends past work by examining the interaction between brain regions tied to the process of divergent thinking. Although the present study benefited from functional connectivity methods, our conclusions regarding the causal relation between brain activity and divergent thinking remain limited. Future work should further examine divergent thinking-related functional connectivity using event-related designs. Such an approach could determine how different neural networks interact during the process of idea generation.

An interesting question for future research to consider is whether highly creative people engage in different thought processes at rest. For example, the creative brain may be more apt to engage in spontaneous imaginative processes (e.g., mind wandering) in the absence of an externally-presented task. If so, this may also explain the resting-state group differences found in the present study; that is, if highly creative people are more likely to engage in imaginative processes, they may also show stronger activity within regions of the default network at rest. One way to test this would be to administer experience-sampling probes in the scanner (cf. Christoff et al., 2009) and ask participants to report on their thought content at random intervals at rest or during a minimally-demanding task that tends to induce mind wandering. Future work could use similar approaches to further shed light on the extent to which controlled and spontaneous processes contribute to creative thought.
